# Spatial and spectrotemporal features of noise alter female responses to costly male signals in Cope’s gray treefrog (*Hyla chrysoscelis*)

**DOI:** 10.1007/s00359-026-01812-6

**Published:** 2026-05-29

**Authors:** Jessica L. Ward, Norman Lee, Lata Kalra, Mark A. Bee

**Affiliations:** 1 Department of Ecology, Evolution, and Behavior, University of Minnesota – Twin Cities, St. Paul, MN USA; 2 Present Address: Department of Biology, Ball State University, Muncie, IN USA; 3 Present Address: Department of Biology, St. Olaf College, Northfield, MN USA; 4 Graduate Program in Neuroscience, University of Minnesota – Twin Cities, Minneapolis, MN USA

**Keywords:** Acoustic communication, Environmental noise, Spatial release from masking (SRM), Comodulation masking release (CMR), Mate choice, Grey tree frog

## Abstract

In anuran choruses, where many males call simultaneously, females must identify attractive conspecific signals amid high levels of background noise. We tested whether spatial separation between signals and noise (spatial release from masking, SRM) and spectrotemporally correlated amplitude fluctuations in noise (comodulation masking release, CMR) influence mate choice behavior and help females maintain preferences for energetically costly calls under noisy conditions. Using two-alternative phonotaxis tests with Cope’s gray treefrogs (*Hyla chrysoscelis*), we measured response probability, the calling effort of chosen males, and response latency in the absence of noise and in six noise treatments that factorially varied the location of the noise and its amplitude modulation pattern. Signals and noise were either colocated or spatially separated, and noise envelopes either lacked modulations (unmodulated) or had modulations that were correlated (i.e., comodulated) or uncorrelated across the frequency spectrum. Females responded in nearly all trials, but selected stimuli with lower calling effort and took longer to choose in the presence of noise. Responses rates were lowest and latencies longest when noise was unmodulated and colocated with signals. Spatially separating noise from signals improved performance across all measures. Introducing amplitude modulations generally yielded higher response rates and shorter latencies, but the impacts of modulation on stimulus selection were more varied, and there was limited evidence that comodulation improved performance. These findings indicate that spatial and spectrotemporal features of noise impact female responses to costly male signals and highlight perceptual mechanisms that may shape the evolution of communication in acoustically complex social environments.

**Supplementary Information:**

The online version contains supplementary material available at https://doi.org/10.1007/s00359-026-01812-6.

## Introduction

Animals frequently communicate using signals that are ostentatious in design and costly to produce (Dawkins and Krebs 1978; Bradbury and Vehrencamp 2011), particularly in the context of mate attraction (Ryan and Keddy-Hector 1992; Rosenthal 2017). A key selective pressure acting on both signalers and receivers is environmental noise, which renders signals more difficult to perceive and which can have profound impacts on the evolutionary design of animal communication systems (Brumm and Slabbekoorn 2005; Brumm 2013; Wiley 2015). For acoustically communicating animals, the noise generated by large social aggregations, such as a colony or chorus of conspecifics, poses particularly significant challenges for effective communication (Klump 1996; Hulse 2002; Bee and Micheyl 2008; Römer 2013; Vélez et al. 2013b). These challenges arise not only because of high signal amplitudes, but also because of the high degree of acoustic similarity between signals of interest (e.g., those of a potential mate) and the "noise" of the aggregation (e.g., the signals of other competing signalers). This similarity is problematic because both signal and noise similarly stimulate the "matched filter" tuning of the receivers' auditory system (Simmons 2013; Henry et al. 2016; Narins and Clark 2016; Römer 2016).

Anuran amphibians (frogs and toads) are particularly well known for their use of acoustic signals in noisy breeding choruses (Narins 1995; Narins et al. 2007). Much of what we know about how frogs hear and communicate in noise stems from pioneering research conducted over the past half century by the honoree of this special issue, Peter Narins, along with his students, postdocs, and collaborators. The immense breadth and depth of Narins’ collective body of work on how frogs hear and communicate in noise have established frogs as neuroethological models for studying sensory ecology and the mechanistic basis of acoustic communication in challenging environments. Narins was among the first to quantify frog chorus noise levels and how they change over the diel cycle (Narins 1982). His electrophysiological studies in the laboratory helped elucidate how the frog auditory periphery encodes signals in noise (Zelick and Narins 1985b; Narins 1987; Dunia and Narins 1989; Narins and Wagner 1989; Penna et al. 2009). His detailed observations and exquisite experiments in the field showed how males can adjust their calling behavior to reduce masking interference (Zelick and Narins 1982, 1983, 1985a; Brush and Narins 1989). His empirical studies led to the fascinating discoveries that frogs use seismic signals (Narins and Lewis 1984; Lewis and Narins 1985; Lewis et al. 2001) and ultrasonic signals (Feng et al. 2006; Arch et al. 2008, 2009; Feng and Narins 2008) to communicate in noisy environments, and Narins was the first to highlight the potential impacts of anthropogenic noise on frogs (Sun and Narins 2005). The present study attempts to extend this inimitable body of work.

We investigated the extent to which spatial and spectrotemporal features of noise potentially impact the responses of female frogs to the costly sexual advertisement signals produced by males in breeding choruses. To this end, we examined the mating behavior and preferences of female Cope’s gray treefrogs (*Hyla chrysoscelis*; AmphibiaWeb 2024) for males producing advertisement calls in the presence of experimentally manipulated “chorus-shaped” noise (i.e., noise with the long-term spectrum of a conspecific breeding chorus). Males of this species and its closely related tetraploid sister species (*Hyla versicolor*) aggregate in suitable breeding habitat and form dense choruses in which they produce pulsatile advertisement calls at high sound pressure levels (~ 85 to 93 dB SPL at 1 m, re 20 µPa, fast RMS) (Gerhardt 1975). Females can use the sounds of a chorus to localize aggregations of signaling males within the available habitat (Swanson et al. 2007; Vélez et al. 2017). Within a chorus, a female selects a mate after recognizing and localizing his advertisement calls. Numerous studies have demonstrated that females of both gray treefrog species prefer males that call at higher rates and that produce longer calls having more pulses, indicating a general preference for higher overall “calling effort” (the product of call rate and the number of pulses per call; Gerhardt 1994; Gerhardt et al. 1996; Bee 2008b; Vélez et al. 2013a; Ward et al. 2013b; Lee et al. 2017; Tanner et al. 2017, 2025; LaBarbera et al. 2020; Li et al. 2022; Krueger et al. 2026). When calling effort is held constant at the population average, females of the diploid species ignore differences in call rate and call duration, thus indicating the importance of calling effort in female mating decisions (Ward et al. 2013b; Krueger et al. 2026). Calling is energetically expensive in gray treefrogs, and males that signal with higher calling effort experience higher energetic demands (Taigen and Wells 1985; Wells and Taigen 1986; Wells et al. 1995). They also have higher levels of circulating testosterone and estradiol (Baugh et al. 2026). Ambient chorus noise poses significant challenges to acoustically guided mate choice by increasing signal recognition thresholds (Bee and Schwartz 2009; Vélez and Bee 2010, 2011, 2013a; Nityananda and Bee 2012; Schwartz et al. 2013), impairing sound pattern recognition (Bee 2008a; Ward et al. 2013a; Lee et al. 2017), constraining the expression of preferences for attractive signals, including those with higher calling efforts (Bee 2008b; Vélez et al. 2013a; Ward et al. 2013b; Lee et al. 2017), and interfering with sound localization (Caldwell and Bee 2014). Unlike mammals and birds (Brumm and Zollinger 2011; Kunc et al. 2022), male gray treefrogs do not display a Lombard Effect by increasing their call amplitude in the presence of noise (Love and Bee 2010), and there is no evidence to suggest male gray treefrogs adjust the timing of their signals to avoid call overlap in dense choruses (Schwartz et al. 2002).

We tested the general hypothesis that two forms of perceptual masking release – spatial release from masking (SRM; Plomp and Mimpen 1981) and comodulation masking release (CMR; Hall et al. 1984) – help female frogs mitigate the challenges of choosing preferred males in noisy choruses. Both SRM (reviewed in Bronkhorst 2000) and CMR (reviewed in Verhey et al. 2003) are well-described phenomena in the literature on human hearing and speech perception in noise. Spatial release from masking occurs when listeners experience improvements in listening performance (e.g., lower signal detection thresholds or better signal recognition) in conditions where sources of signals and noise are perceived as being spatially separated. Comodulation masking release refers to improvements in performance that listeners experience when temporal modulations in noise amplitude are statistically correlated in time (i.e., “comodulated”) across the frequency spectrum. Studies of humans using simple sounds (e.g., tones and narrowband noise) indicate that spatial separation (or binaural disparities) and temporal modulation patterns can have additive effects on masking release or interact to produce either sub-additive or supra-additive effects, depending on several stimulus-related factors (Hall et al. 1988, 2011; Buss et al. 2003; Epp and Verhey 2009a, b; Middlebrooks 2017). In the context of human speech, both phenomena contribute to solving the “cocktail party problem” (Cherry 1953; Bronkhorst 2000; McDermott 2009; Middlebrooks et al. 2017), an anthropocentric label for the more widespread biological problem of communicating acoustically in noisy social groups (Bee and Micheyl 2008). However, the extent to which these two perceptual phenomena enable nonhuman receivers to solve similar communication problems has received much less attention.

In previous studies of SRM and CMR, we have shown that female Cope’s gray treefrogs can partially mitigate the negative impacts of noise by taking advantage of spatial separation between sources of signals and noise (Bee 2007, 2008a; Nityananda and Bee 2012; Ward et al. 2013a; Caldwell et al. 2016), slow temporal fluctuations in the amplitude of background noise (Vélez and Bee 2010, 2011, 2013), and comodulation across the frequency spectrum of ambient noise (Lee et al. 2017; Bee and Vélez 2018). In the present study, we conducted two-alternative choice tests to examine the potential impacts and interaction of SRM and CMR on a female’s choice of males differing in calling effort. Across treatments, females chose between two simulated males with different calling efforts in the presence of chorus-shaped noise consisting of two distinct spectral bands separately encoded by the two physically distinct sensory papillae in the frog inner ear responsible for transducing airborne sound. Noise was presented from positions that were either colocated with, or spatially separated from, signals, and its two spectral bands had a temporal amplitude structure that was either unmodulated, comodulated (i.e., correlated across bands), or independently modulated (i.e., uncorrelated across bands). We evaluated the separate and joint effects of noise location and noise modulation on response rates, the calling efforts of chosen males, and the latency females required to make their choice.

## Materials and methods

### Subjects

We conducted phonotaxis tests with gravid female *H. chrysoscelis* from the western genetic lineage (Booker et al. 2022) in May and June 2013. Subjects were collected in amplexus between 2200 and 0100 h from Carver Park Reserve (Carver County: 44°52′32.76"N, 93°41′5.58"W), Crow-Hassan Park Reserve (Hennepin County: 45°10′52.1"N, 93°38′07.3"W) and Lake Maria State Park (Wright County: 45°19′11.51"N, 93°56′37.67"W). Breeding pairs were kept moist and transported to the lab in separate plastic containers where they were maintained at approximately 4 °C until the day of testing to prevent egg deposition (Gerhardt 1995). Approximately 30 min prior to testing, we placed pairs in an incubator to bring their body temperatures to 20 ± 1 °C, the temperature at which behavioral tests were conducted. We separated subjects from their mates during tests but returned them to their mates in the incubator between tests and after testing was finished. All pairs were returned to their capture site within five days of collection. All procedures for the collection, handling and testing of frogs were approved by the University of Minnesota Institutional Animal Care and Use Committee (#1202A10178).

### Hypotheses and experimental design

**Fig. 1:**
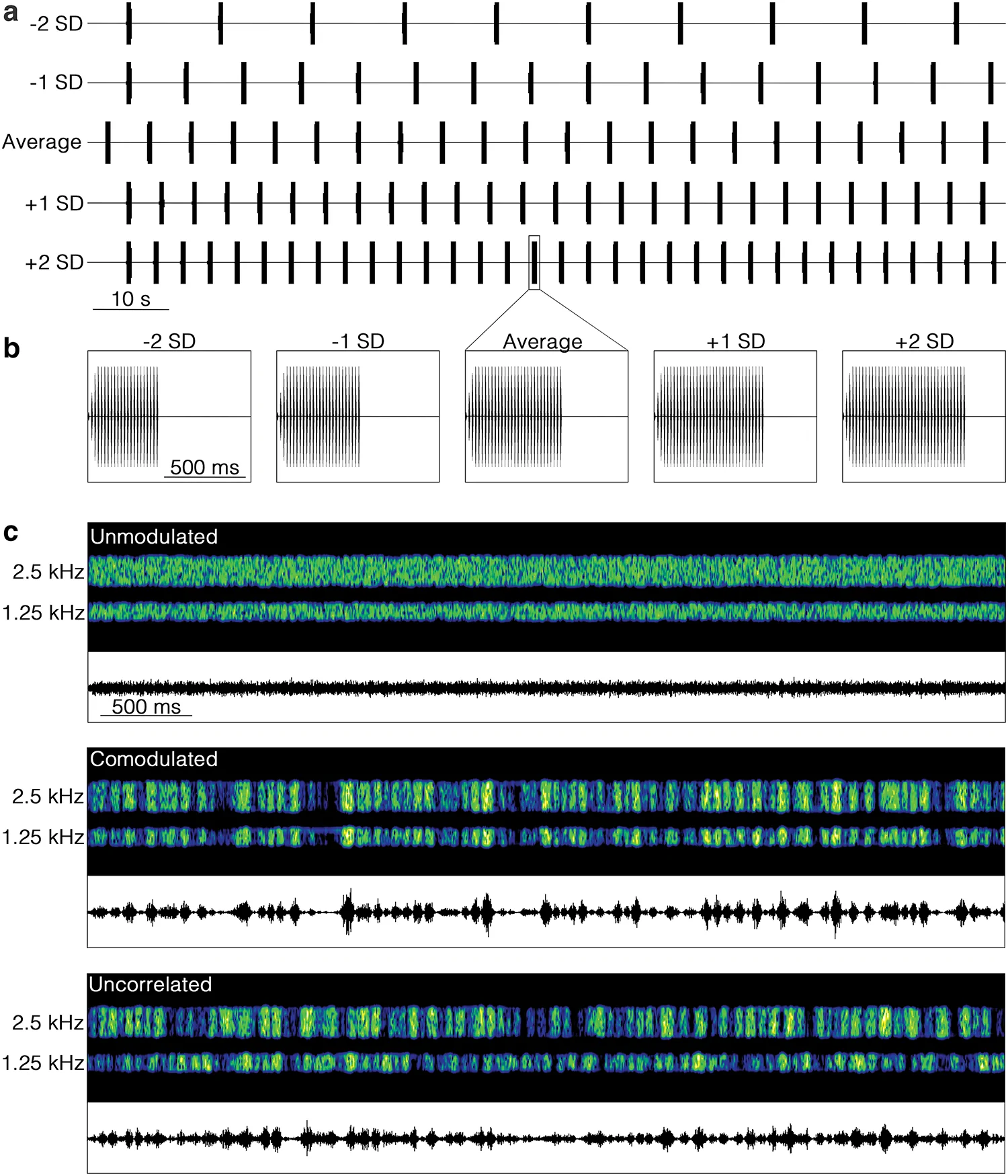
Acoustic stimuli. **a** Waveforms showing 2 min of the sequences of synthetic calls used to examine female preferences for calling effort based on manipulating call rate. In each two-alternative choice test, the average call (11 calls/min) was paired against an alternative with a call rate that differed in units of standard deviation (SD): -2 SD (5 calls/min), -1 SD (8 calls/min), + 1 SD (14 calls/min) or + 2 SD (17 calls/min). The sequences depicted here show the average call in the leading position and illustrates how calls drifted in and out of phase between the two paired alternatives according to differences in call rate. **b** Waveforms showing the synthetic calls used to examine female preferences for calling effort based on manipulating call duration. In each two-alternative choice test, a sequence of average calls (30 pulses/call) was paired against an alternative with a call duration that differed from the mean in SD units: -2 SD (22 pulses/call), -1 SD (26 pulses/call), + 1 SD (34 pulses/call) or + 2 SD (38 pulses/call). **c** Spectrograms (*top*, 512 pt FFT) and waveforms (*bottom*) showing 5 s of the unmodulated, comodulated, and uncorrelated noises used in two-alternative choice tests conducted in noise

Our experimental design was based on the a priori expectation that females prefer males that signal with relatively higher calling efforts (i.e., faster call rates or longer call durations), as they are well known to do when tested in the absence of noise (Gerhardt 1994; Gerhardt et al. 1996; Bee 2008b; Vélez et al. 2013a; Ward et al. 2013b; Lee et al. 2017; Tanner et al. 2017, 2025; LaBarbera et al. 2020; Li et al. 2022; Krueger et al. 2026). We tested the hypothesis that SRM and CMR improve performance in mate choice decisions in the presence of noise, including the ability of females to exercise preferences for males with higher calling efforts. This hypothesis generally predicts higher response rates, higher calling efforts among chosen males, and shorter response latencies when signals and noise are spatially separated and when signals are presented with comodulated noise compared with unmodulated and uncorrelated noise (Fig. 1). By using a factorial design, we also attempted to test the hypothesis that SRM and CMR operate independently, and thus have additive effects, to determine the magnitude of masking release versus the alternative hypothesis that they interact and produce either sub-additive or supra-additive effects.

To test our predictions, we conducted a series of 8 two-alternative choice tests in which we manipulated calling effort (Table S1). Because any single value of calling effort can be substantiated through numerous combinations of call rate and call duration (i.e., the number of pulses per call), we elected to manipulate calling effort by specifically varying either call rate (four tests) or call duration (four tests), the two components that together establish a male’s calling effort (Ward et al. 2013b; Krueger et al. 2026). Each of the eight tests was replicated once in the absence of noise and once in each of six noise conditions consisting of the 2 × 3 factorial combinations of two noise locations (colocated and separated) and three noise modulation types (unmodulated, comodulated, and uncorrelated). In each test, we presented the subject with two sequences of synthetic calls that simulated two calling males with different calling efforts (based on differences in call rate or call duration) but that were otherwise identical in their spectral and fine temporal properties (Fig. 1a, b). One stimulus in each test was an “average call” and had a call rate of 11 calls/min, a call duration of 30 pulses/call, and consequently a calling effort of 330 pulses/min (30 pulses/call × 11 calls/min). These values are close to the average values at 20 ºC for our study population (mean ± SD call rate: 11.4 ± 2.9 calls/min; call duration: 30 ± 4 pulses/call; calling effort: 333 ± 126 pulses/min; Ward et al. 2013b). The alternative stimulus in each test differed from the average call in the property of interest by either ± 1 SD or ± 2 SD, where 1 SD in call rate was 3 calls/min and 1 SD in call duration was 4 pulses/call. Initial analyses of the effects of noise modulation (and only noise modulation) on the binary outcome of choice tests conducted with ± 2 SD differences were reported in Lee et al. (2017). These data are included in the present study to investigate the effects of noise modulation, as well as noise location and their potential interaction, on different response variables – response probability, chosen calling effort (in pulses/min), and response latency – using a richer set of stimulus manipulations (± 1 SD and ± 2 SD).

In the four tests in which the call rates of the two stimuli differed, we held call duration constant at the population average (30 pulses/call) and systematically manipulated the repetition rate of the alternative call across the four tests (5, 8, 14, or 17 calls/min; Fig. 1a), such that the four alternative calls had calling efforts of 150, 240, 420, and 510 pulses/min, respectively (Table S1). In the four tests in which call duration differed between the average and alternative calls, we held call rate constant at the population average (11 calls/min) and manipulated call duration by varying the number of pulses in the alternative call (22, 26, 34, or 38 pulses/call; Fig. 1b), such that the four alternative calls had calling efforts of 242, 286, 374, and 418 pulses/min, respectively (Table S1). In all tests, the rates and durations of alternative calls fell within the ranges of variation in individual means previously recorded in our study population (call rate: 6.1–19.9 calls/min; call duration: 22–38 pulses) (Ward et al. 2013b). Across all eight tests, the differences in calling effort between the average and alternative stimuli expressed in SD units were ± 0.35, ± 0.70, ± 0.71, ± 1.42 SD. We tested different groups of subjects in each of the 8 two-alternative choice tests (*n* = 30/test; total *n* = 240 subjects). Each subject was tested seven times, once with its designated choice in the absence of noise and once in each of the six factorial combinations of noise location and noise modulation. Test order was randomized separately for each subject.

### Synthetic calls and chorus-shaped noise

We used MATLAB^®^ 7.6.0 (MathWorks, Natick, MA, USA) to generate synthetic stimulus calls (WAV, 44.1 kHz sampling rate, 16-bit resolution). Each call was composed of a train of identical pulses, each of which was 10 ms in duration and separated by 10-ms inter-pulse intervals (20-ms pulse period; 50 pulses/s). An individual pulse was created by adding two harmonically related, phase-locked sinusoids with frequencies (and relative amplitudes) of 1.25 kHz (− 9 dB) and 2.5 kHz (0 dB). We shaped the pulse to have a species-specific amplitude envelope, with a rise time of 3.6 ms and a fall time of 6.4 ms. Calls differing in duration were created by concatenating pulses and inter-pulse intervals until the desired number of pulses per call was achieved. For each stimulus call, we shaped the call amplitude envelope using a linear rise over the first 60 ms of the call. We concatenated calls of appropriate lengths and call periods until sound files just exceeded 6 min in total length.

Tests were conducted in the absence of noise and in the presence of synthetic, chorus-shaped noise (Fig. 1c) that lacked temporal modulations in amplitude (unmodulated) or had temporal modulations in amplitude that were either correlated (i.e., comodulated) or uncorrelated across frequency. The unmodulated noise had a steady state temporal envelope that did not fluctuate beyond the inherent fluctuations present in narrowband noise. We generated eight exemplars of unmodulated noise using eight different white noises. For each exemplar, unmodulated noise was created by bandpass filtering white noise between 1050 and 1450 Hz and between 2100 and 2900 Hz to result in two narrowband noises, one 400 Hz wide and a second 800 Hz wide, centered at 1250 and 2500 Hz, respectively (Fig. 1c). These two noise bands effectively mimic the spectrum of natural gray treefrog choruses (Vélez et al. 2017) and fall in frequency ranges that are transduced, respectively, by the gray treefrog amphibian papilla and basilar papilla, which are the two sensory papillae in the frog inner ear sensitive to airborne sounds (Hillery 1984; Schrode et al. 2014). Temporally modulated chorus-shaped noises were created by first imposing the envelope of a narrowband noise modulator on the same eight white noises used to generate exemplars of unmodulated noise followed by bandpass filtering the resulting modulated white noises into the same two bands present in the unmodulated noise. We created narrowband noise modulators by applying a lowpass filter with a cutoff frequency of 12.5 Hz to different white noises. To create comodulated noise, the same noise modulator was used to modulate both noise bands of chorus-shaped noise so that the resulting noise exhibited temporal envelope fluctuations that were correlated across the frequency spectrum (Fig. 1c). In uncorrelated chorus-shaped noise, the two noise bands were modulated with different noise modulators, resulting in temporal envelope fluctuations in the lower frequency noise band that were independent of fluctuations in the higher frequency noise band (Fig. 1c). Each set of exemplar chorus-shaped noises was created with equivalent RMS amplitudes. Each subject was tested with the same set of exemplars, and different groups of subjects were tested with different exemplar sets; preliminary analyses indicated no systematic differences in responses dependent on noise exemplars.

### Apparatus and testing protocol

**Fig. 2:**
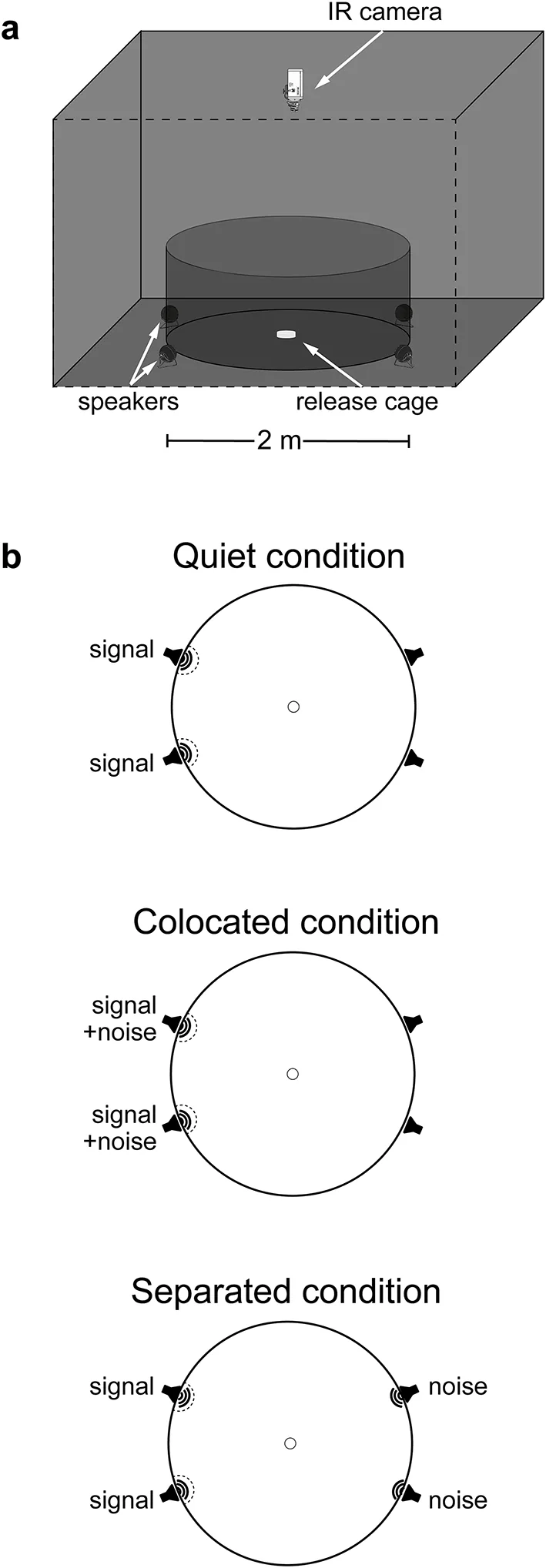
Experimental apparatus. **a** Schematic diagram of the test arena inside the sound chamber. **b** Overhead schematic view of the test arena showing the two pairs of speakers through which signals and noise were broadcast in quiet and the colocated and separated noise conditions. The two speakers in each pair were separated by 45°. The two pairs of speakers were placed on opposite sides of the arena such that each speaker within a pair was separated from the two speakers in the opposite pair by 135° and 180°, respectively

We conducted tests under infrared (IR) light in a temperature-controlled (20 ± 1 °C), semi-anechoic sound chamber (Fig. 2a; 3.0 m × 2.8 m × 2.2 m, L × W × H; Industrial Acoustics Company, IAC, North Aurora, IL, USA). The walls and ceiling were lined with acoustic insulation covered with dark grey perforated metal (IAC’s Planarchoic™ panel system). The floor of the sound chamber was covered in dark grey, low pile carpet. In the center of the chamber was a 2-m diameter circular test arena bordered by a 60-cm high wall made of hardware cloth and covered in acoustically transparent but visually opaque black fabric (Fig. 2a). On the floor in the center of the test arena was a 9-cm diameter (2-cm height) acoustically transparent release cage that could be operated from outside the chamber. We broadcast sounds from two pairs of speakers (Mod1 Orb speaker, Orb Audio, New York, NY, USA) positioned on the floor outside the perimeter of the arena wall and facing inward toward the arena’s center (Fig. 2b). We marked a 10-cm semicircular response zone in front of each speaker for one of the pairs. Subject responses were scored during tests using a monitor located outside the chamber and connected to an IR-sensitive Panasonic WV-BP334 video camera (Panasonic Corporation of North America, Secaucus, NJ, USA) positioned inside the chamber at ceiling height directly above the center of the test arena.

We broadcast sounds using Adobe Audition 1.5 (Adobe Systems Inc., San Jose, CA, USA) running on a Dell Optiplex 980 PC computer (Dell Computer Corporation, Round Rock, TX, USA) located outside of the chamber. Sounds were output through an M-Audio FireWire 410 multichannel soundcard (M-Audio, Irwindale, CA, USA) and amplified using a HTD DMA-1275 amplifier (Home Theater Direct, Inc., Plano, TX, USA). In all choice tests, the first three calls alternated exactly out of phase with respect to their midpoints, such that the second call broadcast was preceded and followed by an equivalent silent interval. In the four tests in which call rate was held constant, this temporal arrangement was maintained for the duration of the test. In the four tests in which call rate differed between the two alternatives, subsequent calls drifted in and out of phase according to their respective call rates (see Fig. 1a). Across subjects, we counterbalanced the relative positions of the playback speakers so that half of the subjects in each test heard the average call coming from either the left or the right speaker in the pair.

At the start of each testing day, and between tests of three to five subjects, we calibrated the sound pressure levels of all calls to 82 dB SPL (LCF_max_) at a distance of 1 m at the subject release site. This amplitude is 3.5 dB below the low end of the range of variation in call amplitudes (fast, RMS) recorded for this species at a distance of 1 m (Gerhardt 1975). For tests conducted in the presence of chorus-shaped noise, the same noise exemplar was broadcast either from the same two speakers as the calls (colocated condition; Fig. 2b) or from the other pair of speakers on the opposite side of the arena (separated condition; Fig. 2b). We separately calibrated the identical noise broadcast from each speaker to be 76 dB (LC_eq_) at the subject release site 1 m from the speaker so that the combined level of both noises, when simultaneously broadcast from both speakers in a pair, was 82 dB at the same position, which falls within the range of sound levels we have recorded in active gray treefrog choruses (Tanner and Bee 2019). This noise level yielded a nominal SNR of 0 dB at the release site in the center of the arena. We chose to test a single SNR of 0 dB for our experiment for three reasons. First, the decision to test a single SNR was a compromise made to implement an otherwise large factorial design given constraints on the number of gravid females available for testing. Second, based on our earlier work with this species, we preferred to use an intermediate SNR that would likely pose challenges to a female without impairing her ability to detect or recognize sounds entirely, as is more likely to occur at SNRs below 0 dB (Bee 2007; Bee and Schwartz 2009; Vélez and Bee 2010). Finally, previous studies have shown SRM and CMR to influence the behavioral responses of female gray treefrogs at this and similar SNRs (Bee 2007, 2008a; Ward et al. 2013a; Lee et al. 2017).

All sound measurements were made using a Brüel and Kjær Type 2250 sound level meter (Brüel and Kjær, Norcross, GA, USA). For calibration, we placed the microphone on a 10-m extension cable at the center of the arena at a distance of 3 cm from the floor and 1 m from each speaker and aimed it at a point on the arena wall directly between the two speakers in a pair that were being calibrated. This was the approximate position of a subject’s head while sitting in the release cage.

To begin each choice test, we placed the subject in the release cage where it was permitted to acclimate for 90 s. For tests conducted in the presence of noise, broadcasts of noise began 60 s into the acclimatization period and continued for the remainder of the test. In all tests, we began broadcasting the alternating signals at the end of the 90-s acclimatization period, and we released subjects after the first 15 s of signal broadcasts. Subjects were given up to 5 min after their release to enter one of the response zones in front of the two speakers broadcasting calls. Subjects that failed to respond within 5 min were scored as ‘no response’. We returned subjects to their mates in the incubator for 5–15 min between consecutive tests. If a subject failed to make a choice in two consecutive tests in the presence of noise, or in its last test, we conducted an additional ‘motivation’ test that consisted of presenting the average call in the absence of noise to assure the subject was still responsive to acoustic stimuli. Subjects that failed to respond in the motivation test were removed from the experiment, and their responses were excluded from the final dataset. We continued testing until 30 subjects had responded in the absence of noise and in each factorial combination of noise location and noise modulation. In total, we tested 255 subjects, 15 of which became unresponsive during testing (final *n* = 240).

### Data analysis

All statistical analyses were conducted in R version 4.4.1 (R Core Team 2024). A significance criterion of ɑ = 0.05 was used for all analyses.

#### Effects of noise presence

We first examined the overall impacts of the presence of noise regardless of its location or modulation structure. To evaluate the effect of noise on response probability while accounting for repeated measures within subjects, we used a generalized estimating equation (GEE) approach with a binomial distribution and a logit link function (*geepack* package, Højsgaard et al. 2016). The model included response (yes/no) as the dependent variable and noise presence (present vs. absent) as a predictor, with subject identity specified as the clustering unit. Tests conducted in the presence of noise were included in this analysis without regard for the location or modulation pattern of the noise. An exchangeable working correlation structure was applied to account for within-subject correlation. Statistical significance was evaluated using Wald χ^2^ tests. To further assess the extent to which noise influenced female choice behavior, we fit two linear mixed-effects (LME) models using restricted maximum likelihood (REML) based on trials in which females responded by making a choice (*lme4* package, Bates et al. 2015). The first model examined differences in the calling effort of the chosen stimulus call (as a continuous variable), and the second examined variation in log_10_-transformed response latency (as a continuous variable). Log transformation of latency data was required to meet assumptions of the model. In both models, noise presence (present vs. absent) was included as a fixed effect, and subject identity was included as a random intercept to account for repeated measures. Significance of fixed effects was evaluated using Type II Wald F tests with Kenward–Roger degrees of freedom (Kenward and Roger 1997). Because stimulus calling effort was strictly ordinal in our experimental design, rather than continuous, we additionally conducted cumulative link mixed-model (CLMM) analyses (see Supplemental Material). These CLMM models incorporated the same fixed and random effects as the LME models and allowed us to evaluate whether inferences regarding calling effort were robust to treating the variable as continuous versus ordinal.

#### Effects of noise location and modulation

We examined the effects of noise location and modulation on response rates, the calling effort of chosen stimuli, and log_10_-transformed response latency in models based on our two location (colocated, separated) × 3 modulation (unmodulated, comodulated, uncorrelated) experimental design. Response probability was modeled using GEE with a binomial distribution and logit link function and included main effects of noise location and modulation and their interaction, with subject identity as the clustering unit. Chosen calling effort (continuous) and log_10_-transformed latency (continuous) were analyzed using LME fitted by REML, with noise location, modulation, and their interaction as fixed effects and subject identity as a random intercept. Significance of fixed effects was assessed using Type II Wald F tests with Kenward–Roger degrees of freedom. Again, we also fit CLMMs that modeled calling effort as an ordinal response variable (see Supplemental Material) to evaluate whether inferences regarding calling effort were robust to treating the variable as continuous versus ordinal. These models used the same fixed‑effects structure and random‑intercept specification as the corresponding LME models.

## Results

### Effects of noise presence

**Fig. 3:**
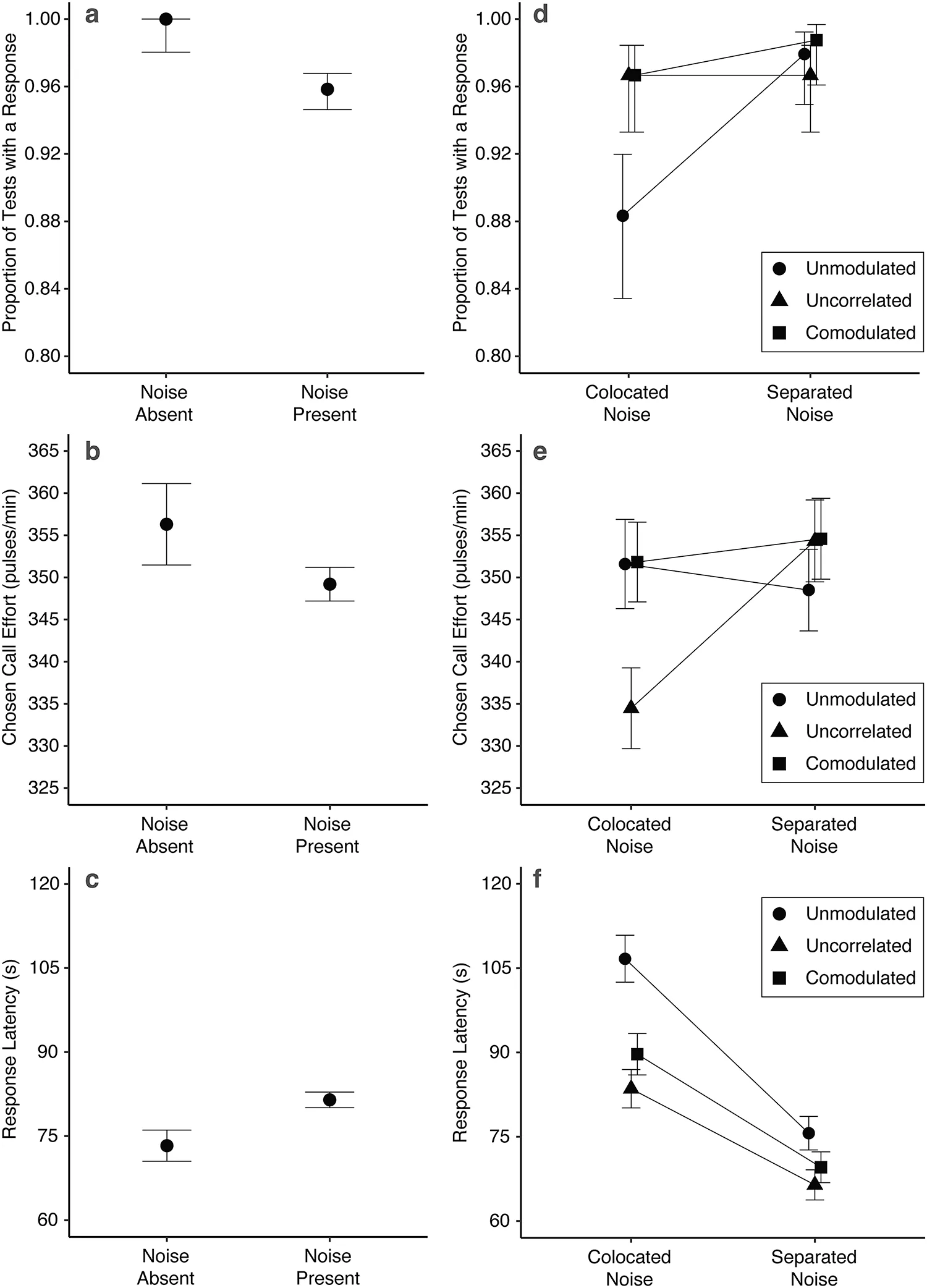
Experimental results. **a–c** Results comparing the effects of noise presence (present, absent) showing **a** the proportion of tests (± 95% exact binomial confidence intervals) in which a response was elicited, **b** the mean (± SE) calling effort of the chosen stimulus and **c** the mean (± SE) response latency for tests in which a response was elicited. **d–f** Results comparing the effects of noise location (colocated, separated) and noise modulation (unmodulated, comodulated, uncorrelated) showing **d** the proportion of tests in which a response was elicited, **e** the mean (± SE) calling effort of the chosen stimulus and **f** the mean (± SE) response latency for tests in which a response was elicited

Overall response rates were high in both the presence and absence of noise, indicating females were able to successfully detect signals, recognize them as conspecific calls, and localize them in the presence of noise at a SNR of 0 dB. Responses were elicited in 100% of trials when noise was absent (240/240; Fig. 3a). In the presence of noise, response rates were significantly lower (β =  − 38.83, SE = 0.13, Wald’s χ^2^ = 96,490, *p* < 0.001) but remained high overall at 95.8% of trials (1380/1440; Fig. 3a). Evidence that females experienced some degree of challenge in the presence of noise was apparent in analyses of chosen calling efforts and response latencies. The mean calling effort of the chosen stimuli was significantly lower when noise was present ($\overline{X }$ = 349 pulses/min) compared to absent ($\overline{X }$ = 356.3 pulses/min) (Fig. 3b; β =  − 8.04, SE = 3.35, F_1,1380_ = 5.74, *p* = 0.017; see Table S2). Response latency was also affected by noise: choices took longer in the presence of noise ($\overline{X }$ ± SE raw latency = 81.5 ± 1.4 s) than in the absence of noise ($\overline{X }$ ± SE 73.3 ± 2.8 s). The model on log_10_-transformed latency confirmed a significant increase in latency when noise was present (Fig. 3c; β = 0.05, SE = 0.01, F_1,1361_ = 10.00, *p* = 0.002). Together, these results indicate that in the presence of noise at a SNR of 0 dB, females were highly responsive but took longer to make a choice and chose stimuli with relatively lower calling efforts.

### Effects of noise location and modulation

Both noise location and noise modulation, as well as their interaction, had significant effects on response rates (Fig. 3d). The lowest response rate (88.3%) occurred in the presence of unmodulated noise presented from a colocated position, whereas response rates in all other conditions exceeded 96% (Fig. 3d). Compared to an unmodulated–colocated baseline, both comodulated noise and uncorrelated noise were associated with higher response rates (β = 1.34, SE = 0.41, Wald’s χ^2^ = 10.62, *p* = 0.001) and separating the noise source further increased response rates (β = 1.83, SE = 0.50, Wald’s χ^2^ = 13.62, *p* < 0.001). A significant interaction indicated that the benefit of noise separation was reduced in the presence of uncorrelated noise (β =  − 1.83, SE = 0.71, Wald’s χ^2^ = 6.62, *p* = 0.010).

There were also significant effects of noise location (F_1,1138_ = 6.55, *p* = 0.011), noise modulation (F_2,1138_ = 4.05, *p* = 0.018), and their interaction (F_2,1138_ = 7.37, *p* < 0.001) on the mean calling effort of chosen stimuli (Fig. 3e; see Table S2). The calling effort of chosen stimuli was lowest ($\overline{X }$ = 334.5 pulses/min) in the presence of uncorrelated noise broadcast from colocated positions and increased by approximately 6% when the same noise was spatially separated ($\overline{X }$ = 354.3 pulses/min) (Fig. 3e). The calling efforts of chosen stimuli were more similar across the other four combinations of noise location and noise modulation (Fig. 3e; 348.5 to 354.6 pulses/min). Notably, there was no evidence that temporal modulations that were correlated across frequency resulted in higher chosen calling efforts: in the colocated condition, chosen calling efforts were nearly identical in comodulated ($\overline{X }$ = 351.8 pulses/min) and unmodulated ($\overline{X }$ = 351.6 pulses/min) noises, and in the spatially separated condition, calling efforts were nearly identical in the comodulated ($\overline{X }$ = 354.6 pulses/min) and uncorrelated ($\overline{X }$ = 354.3 pulses/min) noises (Fig. 3e).

An analysis of log_10_-transformed response latencies showed significant effects of noise location (F_1,1124_ = 138.5, *p* < 0.001) and modulation (F_2,1121_ = 23.6, *p* < 0.001), with a marginal interaction (F_2,1120_ = 2.84, *p* = 0.059). Females were slower to make choices when signals and noise were colocated (range of means across noise modulations: 83.5 s to 106.7 s) compared to the spatially separated conditions (range: 66.4 s to 75.6 s)(Fig. 3f). Across both noise locations, responses were faster in the presence of modulated noise compared with unmodulated noise; however, the effects of differences in noise modulation were more pronounced in the colocated condition (Fig. 3f), where the presence of modulation reduced response latencies by 18.8% (comodulated: $\overline{X }$ = 89.7 s; uncorrelated: $\overline{X }$ = 83.5 s) compared with the unmodulated condition ($\overline{X }$ = 106.7 s). Response latencies were shortest when uncorrelated noise was spatially separated ($\overline{X }$ = 66.4 s).

## Discussion

Numerous studies across diverse species have investigated how selection on signalers, in terms of optimal signal design (e.g., Brenowitz 1982; Ryan and Brenowitz 1985; Narins et al. 2004; Brumm and Naguib 2009) and signaling plasticity (Brumm and Todt 2002; Brumm et al. 2004; Gross et al. 2010; Holt and Johnston 2014; Kunc et al. 2022), has operated to mitigate the challenges of communicating acoustically in noise. By comparison, fewer studies of animals have investigated the mechanisms, beyond the spectral and temporal selectivity of the auditory system, by which receivers may be adapted to mitigate the impact of noise (Klump 1996, 2016; Bee 2012, 2015; Römer 2013, 2016; Vélez et al. 2013b). Our aim in the present study was to test the hypothesis that SRM and CMR improve the performance of female Cope’s gray treefrog in choosing a mate and exercising preferences for males with higher calling efforts in the presence of chorus-shaped noise. Although females were significantly less likely to respond in the presence of noise, they still maintained a high level of responsiveness in conditions with noise. However, noise resulted in longer response latencies and choices of simulated males with relatively lower calling efforts compared to tests conducted in the absence of noise. This pattern of results indicates that our choice of 0 dB as the single SNR created listening conditions in which females experienced some perceptual challenges yet were still able to choose a mate.

The present study extends our current understanding of SRM and CMR in anurans by showing that noise location and modulation structure can jointly shape female performance, but their contributions were cue-dependent and measure-specific in ways that were not entirely predictable. Spatial release from masking conferred relatively large gains across measures when signals and noise were spatially separated. At our nominal SNR of 0 dB, response rates (cf. Figure 3a, d) and latencies (cf. Figure 3c,f) in the spatially separated conditions were similar to those observed in the absence of noise, suggesting potential ceiling effects for these two response measures when signals and noise were spatially separated. By ceiling effects, we mean performance in the presence of spatially separated noise at an SNR of 0 dB was similar to performance when noise was absent. These ceiling effects limited our ability to probe the impacts of comodulation in spatially separated conditions. Consistent with this view, the benefits of temporal modulation were most apparent in the colocated conditions. When signals and noise were colocated, females responded least often and most slowly in unmodulated noise. Introducing temporal modulations in colocated noise increased response rates to near 100% and reduced response latencies by almost 20%. Both comodulated and uncorrelated modulation patterns improved performance in the colocated condition (i.e., in the absence of spatial cues) to similar degrees. By contrast, when signals and noise were spatially separated, response rates approached 100% across all modulation treatments, and latencies were uniformly shorter, again with only small differences between comodulated and uncorrelated noise. A somewhat different pattern emerged for chosen calling effort, for which the worst performance (i.e., lowest chosen calling efforts) occurred when the noise was colocated with signals and had an uncorrelated modulation structure. Chosen calling efforts were higher and more similar in all other conditions. There was no clear pattern indicating females chose males with higher calling efforts because of spectrotemporally correlated modulation patterns, per se. The statistically significant interactions between noise location and modulation were due to the fact that noise modulation effects were larger in colocated conditions compared with spatially separated conditions. However, the ceiling effects observed in spatially separated conditions made it difficult to determine whether SRM and CMR together have additive, sub-additive, or supra-additive effects. Additional tests at other SNRs (e.g., − 3 dB or − 6 dB) might have avoided these ceiling effects and thus might have revealed additive or interactive effects of SRM and CMR. While it was a necessary compromise in study design, testing a single SNR is an important limitation of the present study that should be addressed in future studies.

Behavioral evidence for SRM in frogs was first reported by Schwartz and Gerhardt (1989), who showed that female green treefrogs (*Hyla cinerea*) experienced approximately 3 dB of masking release when sources of calls and broadband noise were spatially separated, though spatial separation did not improve discrimination between advertisement and aggressive calls. Subsequent work in *H. chrysoscelis* has demonstrated somewhat larger and more functionally relevant benefits of spatial separation, including faster phonotaxis and lower recognition thresholds when signals and chorus-shaped noise were separated (Bee 2007; Nityananda and Bee 2012), as well as improved discrimination of signal patterns such as the differences in pulse rate between conspecific versus heterospecific calls (Bee 2008a; Ward et al. 2013a). Biophysical and neurophysiological studies complement these behavioral findings and implicate both peripheral and central mechanisms in SRM. Caldwell et al. (2016) showed in *H. chrysoscelis* that SRM is mechanistically supported by the frog’s internally coupled ears, which act as inherently directional pressure-gradient receivers (reviewed in Bee and Christensen-Dalsgaard 2016). Electrophysiological recordings from auditory nerve fibers and midbrain neurons in northern leopard frogs, *Rana pipiens*, revealed greater SRM at the level of the midbrain than the auditory periphery, suggesting additional central processing beyond the inherent directionality of the ears (Ratnam and Feng 1998; Lin and Feng 2001). This hypothesis was strengthened by evidence that pharmacologically reducing binaural inhibition in the midbrain diminishes neural SRM to levels comparable to those seen in auditory nerve fibers, indicating an important role for inhibitory binaural interactions in SRM (Lin and Feng 2003). These results support the view that SRM emerges from the interaction between peripheral directionality and central binaural processing in frogs. Collectively, results from the present study, along with previous behavioral and neural evidence, converge on the conclusion that SRM is likely a robust and ecologically relevant mechanism supporting acoustic communication in frog choruses, while also highlighting the need for integrative studies linking peripheral mechanics, central processing, and perceptual outcomes across species and tasks.

In contrast, the present study and previous behavioral studies provide mixed evidence that treefrogs can also exploit comodulation in noise. The noise generated by frog choruses exhibits species-specific temporal modulations that are correlated across the frequency spectrum (Vélez and Bee 2010; Lee et al. 2017; Vélez et al. 2017), confirming the ecological availability of spectrotemporal modulation in natural noise. However, across studies conducted to date, modulation-related performance benefits are task- and species-dependent, and they appear to be more limited than benefits due to spatial separation between signals and noise. Several studies have used temporally fluctuating broadband noise (e.g., 850–3300 Hz or 500–4500 Hz) presented from an overhead speaker to investigate the extent to which females benefit from “acoustic glimpses” of signals in amplitude-modulated noise via so-called “dip listening” (Buus 1985; Cooke 2006). For example, Vélez and Bee (2010, 2011) showed that female gray treefrogs experienced a modest (2–4 dB) masking release in slowly fluctuating sinusoidally amplitude modulated (SAM) broadband maskers, with no benefit at intermediate rates and additional masking at fast rates that overlapped the pulse rate of conspecific calls. No benefit of temporal modulation was observed under similar conditions in two-choice tests in which females discriminated between stimuli differing in calling effort based on differences in call duration (Vélez et al. 2013a). Females of *H. cinerea* showed little evidence of dip listening when tested under similar conditions (Vélez et al. 2012), and neither treefrog species experienced performance improvements when broadband noise had the natural amplitude fluctuations typical of real choruses (Vélez and Bee 2013; Vélez et al. 2013a). In these previous behavioral studies, the modulated noises used were comodulated across frequency, and no uncorrelated condition was incorporated to specifically investigate CMR.

Studies specifically of CMR in gray treefrogs have used multiple narrowband noises to show that performance benefits can emerge when their amplitude envelopes are comodulated (Lee et al. 2017; Bee and Vélez 2018). For example, females achieved lower signal recognition thresholds and made fewer errors in a species discrimination task under comodulated versus uncorrelated or unmodulated maskers. However, the performance benefits in an intraspecific discrimination task were more modest: the probability of choosing a simulated male with a higher calling effort were slightly but significantly higher in comodulated noise compared to uncorrelated and unmodulated noise (Lee et al. 2017). This result departs from the present study, in which the worst call discrimination performance was observed under uncorrelated noise in the colocated condition, and performance in comodulated and unmodulated noise was similar. The present study built on the results of Lee et al. (2017) in controlling for the effects of spatial separation and including more levels of difference in calling effort. At present we have no mechanistic explanation for these observed differences across studies. We speculate that the negative impacts of uncorrelated noise observed in the present study may be related to frequency channel-dependent temporal processing of call features, as has been reported in the tetraploid gray treefrog (Reichert and Höbel 2017). Noise that fluctuates independently at different frequencies might produce additional interference to the extent that temporal features related to calling effort are processed in channel-dependent ways. Answering this question, as well as exploring observed differences across studies, will require additional research. To date, only a single neurophysiological study has investigated the effects of temporal noise modulation on sound processing in frogs. In *R. pipiens*, Goense and Feng (2012) identified subsets of midbrain neurons that show reduced masking in modulated noise, enhanced responses during envelope dips, and sensitivity to masker bandwidth and modulation, findings consistent with neural contributions to dip listening and CMR.

In summary, current evidence from this and previous studies suggests spatial release from masking and temporal unmasking (dip listening and possibly CMR) potentially operate as complementary strategies for mitigating acoustic interference in the context of frog communication, with temporal cues most beneficial when spatial cues are constrained. Important questions remain as to how these processes play out in the natural acoustic scene of a noisy breeding chorus. The experimental noises used in this study attempted to incorporate several key elements of biological realism that reflect the noise of conspecific choruses (e.g., sound pressure level, spectral content, amplitude modulation, spectrotemporal correlation). But there are limits as to how well such experimental noises can emulate the natural “noise” of a frog chorus, which is generated by multiple, simultaneously active sources spread out across two or three physical dimensions within the available habitat. Additional work that incorporates even greater biological realism will be needed to determine the extent to which SRM, CMR, and dip listening benefit frogs in the noisy conditions present in natural breeding choruses. The present study also leaves unanswered the important question of whether the observed impacts of spatial separation and spectrotemporal modulation on response probability, stimulus selection, and response latency translate into evolutionarily functional consequences for signalers and receivers.

While comparative studies across other vertebrates and invertebrates also demonstrate that nonhuman animals are adapted to exploit spatial and spectrotemporal structure in noise to alleviate auditory masking, comprehensive treatments of these mechanisms in the contexts of acoustic communication remain relatively rare outside of work on frogs. Evidence for SRM has also been reported in echolocating bats (Sümer et al. 2009), songbirds (Dent et al. 2009), and orthopteran insects (Schmidt and Römer 2011). Likewise, sensitivity to spectrotemporal correlations in noise, as expressed through CMR, has been demonstrated in behavioral or electrophysiological studies of grasshoppers (Ronacher and Hoffmann 2003), goldfish (Fay 2011), starlings (Klump and Langemann 1995; Klump and Nieder 2001; Hofer and Klump 2003; Bee et al. 2007; Langemann and Klump 2007) mice (Klink et al. 2010), gerbils (Diepenbrock et al. 2017), guinea pigs (Pressnitzer et al. 2001), cats (Nelken et al. 1999), and dolphins (Branstetter and Finneran 2008). Within this broader comparative landscape, frogs have emerged as a uniquely tractable system for studying the perceptual benefits of SRM and CMR, and auditory scene analysis more broadly, across behavioral, physiological, and ecological levels of analysis (Bee 2012, 2015; Vélez et al. 2013b; Lee et al. 2023). Work on anurans has linked natural chorus acoustics, peripheral specializations, central auditory processing, and decision-making in communication, revealing how spatial and spectrotemporal cues can jointly shape performance under adverse masking conditions that simulate several key elements of biological realism. This integrative approach reflects the enduring legacy of Peter Narins, whose pioneering contributions to anuran bioacoustics and auditory neuroethology helped establish frogs as model organisms for understanding how vertebrate sensory systems operate in real-world environments.

## Supplementary Information

Below is the link to the electronic supplementary material.

Supplementary Material 1

## Data Availability

All data supporting the findings of this study are available within the paper and its Supplementary Material.
